# Are Standard Follow-Up Parameters Sufficient to Protect Neurocognitive Functions in Patients with Diabetes Mellitus who Underwent Coronary Artery Bypass Grafting?

**DOI:** 10.21470/1678-9741-2019-0160

**Published:** 2020

**Authors:** Hakan Sacli, Ibrahim Kara

**Affiliations:** 1 Department of Cardiovascular Surgery, Sakarya University Medical Faculty, Sakarya, Turkey.

**Keywords:** Cardiopulmonary Bypass, Cognitive Dysfunction, Spectroscopy, Near-Infrared, Coronary Artery Bypass, Diabetes Mellitus

## Abstract

**Objective:**

We aimed to compare the effectiveness of cognitive function protection between the standard follow-up parameters and advanced neuromonitoring methods in diabetic patients who underwent coronary artery bypass grafting during cardiopulmonary bypass.

**Methods:**

Study design was prospective and observational. Patients were separated into two groups, treated only with standard follow-up parameters (Group 1) and followed up with the change of regional cerebral tissue oxygenation (rSO_2_) by near-infrared spectroscopy (Group 2). Neurocognitive functions were evaluated preoperatively and postoperatively before discharge in all patients using the Montreal Cognitive Assessment (MoCA) test.

**Results:**

Cognitive functions of Group 2 patients in the postoperative period were significantly higher than Group 1 patients (*P*=0.001). The mean postoperative MoCA score of patients was significantly lower than the mean preoperative MoCA score in Group 1 (24.8±2.2 *vs*. 23.6±2.6, *P*=0.02). However, mild cognitive dysfunction was significantly lower in Group 2, compared to Group 1 (*P*=0.02).

**Conclusion:**

In patients followed up with standard parameters, a significant decrease in cognitive function was observed in the early period. However, the use of advanced neuromonitoring methods can significantly prevent this decrease in cognitive functions.

**Table:** 

Abbreviations, acronyms & symbols				
CABG	= Coronary artery bypass grafting		MoCA	= Montreal Cognitive Assessment
CPB	= Cardiopulmonary bypass		NIRS	= Near-infrared spectroscopy
DM	= Diabetes mellitus		O_2_Hb	= Oxyhemoglobin
EAU	= Epiaortic ultrasound		PCO_2_	= Partial carbon dioxide
FiO_2_	= Fraction of inspired oxygen		pO_2_	= Partial oxygen
Hb	= Hemoglobin		POCD	= Postoperative cognitive dysfunction
HCT	= Hematocrit		rSO_2_	= Regional cerebral tissue oxygenation
HHb	= Deoxyhemoglobin		SPSS	= Statistical Package for the Social Sciences
MAP	= Mean arterial blood pressure		TEE	= Transesophageal echocardiography

## INTRODUCTION

Although the improvement and applicability of surgical techniques in open heart surgery have positive gains, there is also an increase in risk factors in the patient population undergoing surgery due to increased average life expectancy. Postoperative cognitive dysfunction (POCD) is seen considerably after major surgeries^[1]^. Its frequency ranges from 10 to 54% and may range from short-term transient forms to postoperative delirium^[2]^. Although it is seen so often, there is no sufficient evaluation and research. Some factors associated with the presence of POCD have been demonstrated. Patients with advanced age, presence of DM and low level of education were reported to be more common in the observed POCD patient groups^[3]^. Diabetes mellitus is one of the major risk factors in atherosclerotic plaque formation and is currently an important health problem which brings additional diabetes-related problems. POCD is one of the diabetes-related problems after major surgeries. The prevalence of DM was 8.8% in 2015 worldwide and this rate is expected to reach 10.4% by 2040, therefore, precautions to be taken are important in this regard^[4]^. Cerebral near-infrared spectroscopy (NIRS) is a system that monitors brain activity and therefore provides information about sufficient cerebral perfusion. Near-infrared region of the electromagnetic spectrum is used in this process (780 nm-2500 nm). It is a technique that measures the amount of absorption of oxyhemoglobin (O_2_Hb) and deoxyhemoglobin (HHb) when the light in near-infrared wavelength passes through the tissues. It gives us values such as cerebral tissue oxygen saturation (rSO_2_). Its normal limits are between 60 and 75% and it is affected by intraoperative cardiac output, blood pressure, PCO_2_, pH, FiO_2_, tissue temperature, hemoglobin concentration and local/regional blood flow^[5]^. Intraoperative rSO_2_ monitoring helps us to interfere with the changes that may result in POCD.

We aimed to compare the effectiveness of cognitive function protection between the standard follow-up parameters and advanced neuromonitoring methods in diabetic patients who underwent coronary artery bypass grafting during cardiopulmonary bypass (CPB).

## METHODS

### Study Population and Exclusion Criteria

Fifty-four patients who underwent isolated and elective coronary artery bypass grafting (CABG) surgery between June and November 2018 were included in the study. Study design was prospective and observational. All patients included in the study had insulin-dependent diabetes mellitus (DM). Onset of diabetes was greater than ten years in all patients and the degree of diabetes was equally in both groups. All patients were assessed after the induction of general anesthesia with intraoperative transesophageal echocardiography (TEE) before sternotomy. After sternotomy, an epiaortic ultrasound (EAU) was performed to assess the level of atherosclerosis to all patients. Modified Wareing classification was used with EAU. The classification level is carried out as 0 (normal), 1 (mild, <3 mm intimal thickness), 2 (medium, ≥3 mm intimal thickness in 1 ascending aorta segment), 3 (severe, ≥3 mm intimal thickness in 2 or 3 aorta segments and frequently accompanying luminal protrusion, ulceration of the mobile structure and plaque surface). All patients have similar EAU results in both groups, but 4 patients included in the study were excluded because calcifications were at level 3. Carotid and vertebral artery Doppler ultrasound were performed in all patients to assess the atheroma condition of the carotid arteries. All patients included in the study had fibrofatty plaque under 2 mm and there was no effect on blood flow in the internal and common carotid artery. Patients were divided into two groups: patients followed up with standard follow-up parameters (Group 1, n=24) and patients whose cerebral tissue saturations (rSO_2_) are followed up with near-infrared spectroscopy (NIRS) in addition to standard parameters (Group 2, n=26). Patients who underwent additional procedure other than CABG, whose EAU showed severe calcification in ascending aorta, with significant carotid artery disease (≥70% of lesion with carotid Doppler USG), very low educational level or illiterate, >80 years old, and with history of cerebrovascular disease, neurovascular seizure and psychiatric disorders, were excluded. The study was approved by the Ethics Committee of Sakarya University Medical Faculty. Informed consent was obtained from all patients included in the study.

### Measurements and Surgical Management

Electrocardiography, heart rate, invasive arterial and jugular venous pressure monitoring, arterial blood lactate level, mean arterial blood pressure (MAP), urinary output, hemoglobin (Hb) and hematocrit (Hct) levels, partial carbon dioxide (pCO_2_) and partial oxygen (pO_2_) with alpha-stat method and peripheral oxygen saturation (SpO_2_) monitoring, routine follow-up parameters, were used in all patients. In addition, intraoperative aortic cross-clamp and total perfusion times, intensive care and hospital length of stay, and regional cerebral oxygen saturation (rSO_2_) parameters were recorded.

**Fig. 1:**
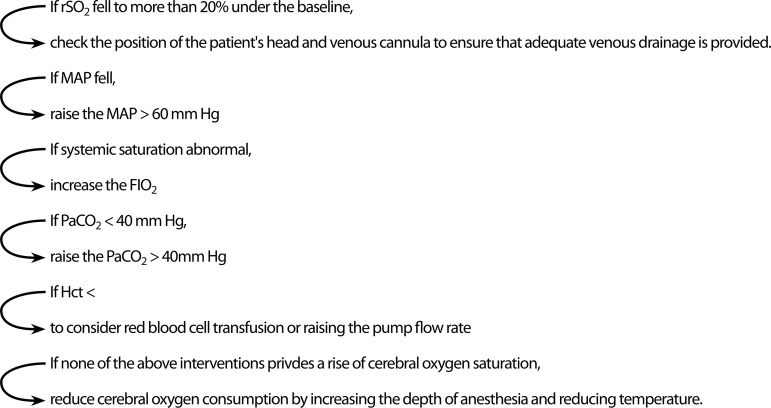
Algorithm for the treatment of cerebral desaturation. FiO_2_=fraction of inspired oxygen; Hct=hematocrit; MAP=mean arterial pressure; PaCO_2_=partial carbon dioxide; rSO_2_=regional cerebral oxygenation

All the surgeries were performed by the same surgery, anesthesia and perfusion teams using the standard surgical protocols and techniques of our clinic. The degree of atherosclerosis in the ascending aorta was evaluated using EAU and digital manual examination. Patients with severe calcific ascending aorta or porcelain aorta were excluded from the study. All patients in Group 2 were monitored with the INVOS system (INVOS 5100C; Somanetics Corp, Troy, MI, USA) to evaluate cerebral perfusion. The probes of the device were placed in both frontotemporal regions reciprocally as the distance between the two probes was approximately 3-4 cm. Prior to anesthesia induction, baseline values were obtained by measuring rSO_2_ values. During CPB in Group 2 patients, right and left rSO_2_ values were recorded continuously. It was considered clinically significant when rSO_2_ values were decreased by >20% compared to baseline values during the procedure and intervention was performed according to the proposed algorithm in the use of brain oximetry (Figure 1)^[6]^.

The neurocognitive functions in all patients were evaluated by physicians preoperatively and postoperatively before discharge by using the Montreal Cognitive Assessment (MoCA) test^[1]^. MoCA test information was given to the patients by our two perfusionists and technically the test was provided by them. This test is an easy and fast screening test that was developed to evaluate mild cognitive functions. The cognitive functions evaluated by the MoCA test include visuopatial/executive, naming, memory, attention, language, abstraction, delayed recall, orientation sections. The highest total score that can be obtained is 30. Accordingly, 26 points and above is normal, 19-25 points are mild cognitive impairment and a score below 19 is considered severe cognitive impairment^[7,8]^. Neurological evaluation included visual and speech impairment, paralysis or weakness in the upper and/or lower extremities, and mental status assessment.

Under nasopharyngeal mild hypothermia (32-34ºC), myocardial protection was provided in all patients with intermittent antegrade cold blood cardioplegia every 15 to 20 minutes, and intermittent antegrade as well as continuous retrograde technique, when necessary. Double clamp technique was performed; distal anastomoses were done under cross-clamp and proximal anastomoses were done under side-clamp, respectively, in all patients. The CPB machine standard flow rate was maintained between 2.2-2.4 L/min/m^2^, pCO_2_ was 35-40 mmHg with the alpha-stat method, MAP was 50-100 mmHg and hematocrit (Hct) was 20%-25% during the operation.

### Statistical Analysis

Data were analyzed with IBM SPSS v23 software. Compliance of data with normal distribution was examined with the Shapiro-Wilk test. Independent samples t-test was used for comparison of data with normal distribution according to groups and paired sample t-test was used for comparison of preoperative and postoperative values in groups. Mann-Whitney U test was used to compare data that are not normally distributed. Chi-square test was used to compare categorical data. While quantitative data showing normal distribution are presented as mean ± standard deviation, data that are not distributed normally are presented as median (min-max). Qualitative data are expressed as frequency (percentage). The significance level was considered as *P*<0.05.

## RESULTS

Demographics and patient data during surgery are presented in Table 1 and Table 2. There was no significant difference between the groups in terms of demographic data. The mortality rate was 0% for both groups. There was no significant change in terms of operating data.

**Table 1:** Description of demographic characteristics.

	Group 1 (n=24)	Group 2 (n=26)	*P*-value
Age (years)	62±7.7	59.8±9.1	0.37
Sex, n (%)			
Male	20 (83.3)	4 (16.7)	0.90
Female	22 (84.6)	4 (15.4)	
Hypertension, n (%)	13 (54.2)	15 (57.7)	0.80
Hyperlipidemia, n (%)	11 (45.8)	9 (34.6)	0.42
Diabetes mellitus, n (%)	10 (41.7)	12 (46.2)	0.75
COPD, n (%)	6 (25)	0 (0)	0.01
CRF, n (%)	2 (8.3)	0 (0)	0.13
Smoking, n (%)	9 (37.5)	12 (46.2)	0.54
Ejection fraction, median (min-max)	55 (40-65)	55 (35-70)	0.61
PAD, n (%)	3 (12.5)	0 (0)	0.06
CVD, n (%)	1 (4.2)	0 (0)	0.29

COPD=chronic obstructive pulmonary disease; CRF=chronic renal failure; CVD=cerebrovascular disease; PAD=peripheral artery disease

**Table 2:** Intraoperative and postoperative data.

	Group 1 (n=24)	Group 2 (n=26)	*P*-value
Number of grafts (mean±SD)	2.5±0.8	2.7±0.5	0.18
CPB time, median (min-max)	83 (40-217)	89 (35-153)	0.52
AC time, median (min-max)	45 (18-170)	50 (15-108)	0.65
Temperature (°C), median (min-max)	30 (28-34)	30 (28-33)	0.39
Major neurological deficit, n (%)	0 (0)	0 (0)	1.00
Mortality, n (%)	0 (0)	0 (0)	1.00
Reoperation for bleeding, n (%)	1 (4.2)	1 (3.8)	0.95
ICU length of stay (days), median (min-max)	3 (1-12)	2 (1-8)	0.04
Length of hospital stay median (min-max)	8 (6-28)	7 (5-16)	<0.01

AC=aortic cross-clamp; CPB=cardiopulmonary bypass; ICU=intensive care unit

### Neurocognitive Results

Since all patients included in the study were diabetic, patients with mild cognitive dysfunction from both groups were present preoperatively. These patients were not excluded because DM is a disease that affects cognitive functions and did not cause a significant difference in terms of between groups preoperative MoCA test. Therefore, the mean preoperative MoCA score of both groups was slightly low (24.8±2.2 *vs*. 25.2±2.0, *P*=0.59) (Table 3). The mean MoCA score and therefore the cognitive functions of patients being monitored for rSO_2_ with NIRS in the postoperative period were significantly higher than those monitored with standard follow-up parameters (23.6±2.6 *vs*. 26.0±2.3, *P*=0.001) (Table 3). The mean postoperative MoCA score of Group 1 patients who were followed with standard follow-up parameters during CABG was significantly lower than the mean preoperative MoCA score (24.8±2.2 *vs*. 23.6±2.6, *P*=0.02) (Table 3). The postoperative and preoperative MoCA scores of patients in Group 2 who were monitored for rSO_2_ with NIRS were similar (25.2±2.0 *vs*. 26.0±2.3, *P*=0.06) (Table 3). Mild cognitive dysfunction was observed in 13 (54.2%) patients in Group 1 and in 5 (19.2%) patients in Group 2. Severe cognitive dysfunction occurred in 2 (8.3%) patients in Group 1, but none in Group 2. When the groups were compared in terms of mild cognitive dysfunction, it was significantly lower in Group 2 (*P*=0.02) (Table 3). Even though severe cognitive dysfunction was more frequently observed in Group 1, it was not significant (*P*=0.22) (Table 3).

**Table 3:** Comparison of cognitive functions between and in the groups.

Comparison of preoperative cognitive results between groups			
	**Group 1 (n=24)**	**Group 2 (n=26)**	***P*-value**
Mild cognitive decline, n (%)	5 (20.8)	6 (23.1)	1.00
Severe cognitive decline, n (%)	0 (0)	0 (0)	1.00
Mean MoCA score	24.8±2.2	25.2±2.0	0.59
**Comparison of postoperative cognitive results between groups**			
	**Group 1**	**Group 2**	***P*-value**
Mild cognitive decline, n (%)	13 (54.2)	5 (19.2)	0.02
Severe cognitive decline, n (%)	2 (8.3)	0 (0)	0.22
Mean MoCA score	23.6±2.6	26.0±2.3	0.001
**Comparison of preoperative and postoperative cognitive results in the groups**			
	**Preoperative**	**Postoperative**	***P*-value**
Group 1, Mean MoCA score	24.8±2.2	23.6±2.6	0.02
Group 2, Mean MoCA score	25.2±2.0	26.0±2.3	0.06

MoCA=Montreal Cognitive Assessment

No major neurological disorder occurred in either group. Intensive care and hospital length of stay were significantly higher in Group 1 compared to Group 2 statistically (*P*<0.01, *P*=0.04, respectively) (Table 2).

## DISCUSSION

The two most important results of this study are; 1) The rate of early postoperative POCD was significantly lower with cerebral rSO_2_ monitoring and intervention in DM patients who underwent CABG compared with the patient group who were followed up with standard follow-up parameters and the POCD rate is demonstrated to be reducible in these patients^[8]^. Interestingly, although the proportion of mild cognitive impairment in the preoperative period in Group 2 has decreased in the postoperative period, despite not being significant, the proportion of mild cognitive impairment in Group 1 has increased significantly in the postoperative period by 3-fold, compared to the preoperative period.

Since the introduction of CPB until today, many improvements have been made in the field of anesthesia and surgery to protect organs and tissues and reduce the complications of CPB, and they still continue^[9]^. The main target for the use of several parameters such as electrocardiography, heart rate, invasive arterial and jugular venous pressure monitoring, arterial blood lactate level, MAP, diuresis, Hb and Hct levels, pCO_2_, pO_2_, SpO_2_, the use of many parameters by anesthesia is to ensure adequate tissue oxygenation during CPB and to maintain this status during the follow-up period^[10,11]^. Although there is a significant decrease in mortality rates with these standard follow-up parameters, there is no significant improvement in POCD rates^[12,13]^. Unfortunately, it cannot be determined whether brain tissue oxygenation, highly susceptible to ischemia especially during CPB, is sufficient or not with the use of standard anesthesia follow-up parameters. Optimal values of routine anesthesia follow-up parameters during CPB, which is an indispensable argument of cardiac surgery, are still controversial. The simplest example is that, although it is indicated that cerebral flow is preserved when MAP is between 50 and 100 mmHg, which is one of the most important follow-up parameters during CPB, its optimal value is still unclear^[14]^. In one study, MAP >70 mmHg and between 50 and 60 mmHg significantly changed the rates of neurological events and, in another study, according to MAP <40 mmHg or >60 mmHg, neurological complication rates were three times different from each other^[15,16]^. This shows us that it is controversial which pressure range will provide optimal cerebral tissue oxygenation. In this study, MAP was maintained between 50 and 100 mmHg in Group 1 patients, whereas in Group 2 patients, intervention was performed according to standard algorithm when cerebral rSO_2_ value decreased >20% compared to baseline.

In the interfered Group 2, POCD rate was three times lower than Group 1 in the early postoperative period. This shows that, in fact, the most ideal MAP value is the MAP value that provides the patient with adequate tissue oxygenation. The incidence of POCD after cardiac surgery varies between 7% and 83% in different studies and it is inevitable that high POCD levels are observed postoperatively^[11,17-19]^. Despite the presence of such high POCD rates, since this condition is not a major neurological deficit, most clinicians may believe that it is transient and they do not take it seriously. Although a high POCD ratio was also observed in our study, no patient developed major neurological deficit. In some studies, it was reported that postoperative POCD was improved in the early period, supporting that POCD was not taken too seriously by clinicians^[20,21]^. However, in many opposing studies, it is reported that the presence of POCD also continues in the long term and disrupts the quality of life and this is an indication that the reality is different^[12,14,17,22,23]^. In a study evaluating the cognitive functions of patients who underwent CABG, the incidence of cognitive decline compared to baseline was reported as 53% at discharge, 36% at 6 weeks, 24% at 6 months and 42% at 5 years^[12]^.

In another study, it was shown that persistent cognitive impairment at 1 year was over 35%^[17]^. In our study, the cognitive impairment rate was 40% in all patients in the early postoperative period (discharge), while it was 62.5% in Group 1 and 19.2% in Group 2. The etiology of decreased postoperative cognitive function is multifactorial and use of CPB was shown as the most important cause of early POCD development^[20]^. While decreased cognitive function makes post-CABG early recovery difficult, recovery is prominent in three quarters of patients in discharge and one third of patients in 6 months^[12]^. One of the most important risk factors for cognitive dysfunction is diabetes mellitus (DM)^[22,24]^. We believe that CPB is the most common cause of POCD in the early postoperative period, and DM is one of the most important risk factors for POCD and cerebral tissue oxygenation and cannot be followed with the standard follow-up parameters during CPB. Optimal values of routine follow-up parameters are controversial, requiring additional monitoring necessary^[14,20,22]^. For this purpose, NIRS monitoring used in our clinic and many clinics is non-invasive, simple to use and can show regional cerebral changes. As far as we know from the literature, there are a few prospective articles in which DM patients are followed up with advanced neuromonitoring during CABG and their cognitive functions are evaluated. According to the results of Colak et al.^[23]^, which is one of these few studies, DM is a strong indicator of prolonged rSO_2_ desaturation during CABG, and prolonged rSO_2_ desaturation is five times more common among diabetic patients compared with non-diabetic patients. DM is a well-known risk factor for the development of coronary artery disease. It is also one of the primary predisposing factors for the development of postoperative neurological disorder after CABG surgery. According to the results of our study, while the rate of postoperative early cognitive impairment was 19.2% in DM patients whose cerebral rSO_2_ is monitored with NIRS during CABG and interfered due to the standard algorithm used by our clinic, this rate in patients who are not followed by NIRS was 62.5%. Cognitive impairment was observed approximately three times less in the intervention group and this is similar to the results of Colak et al.^[23]^. Cognitive outcomes in the early postoperative period were worse in diabetic patients compared to non-diabetic patients and it was associated with failure to optimize perfusion strategies during CPB^[25]^.

The low number of patients in both groups was one of the limitations of our study. Another limitation was that there were patients with mild cognitive dysfunction according to the preoperative MoCA test in both groups. There was no patient with severe dysfunction. However, since there was no significant difference between the two groups in terms of preoperative cognitive functions, this situation did not affect the results.

## CONCLUSION

The results of our study show that standard follow-up parameters can be optimized according to the patient during CABG surgery, with following cerebral regional tissue oxygenation that can decrease cognitive dysfunction, positively affecting the results. Our results need to be supported by randomized trials with a high patient population.

**Table:** 

Authors' roles & responsibilities	
HS	Substantial contributions to the conception or design of the work; or the acquisition, analysis, or interpretation of data for the work; drafting the work or revising it critically for important intellectual content; agreement to be accountable for all aspects of the work in ensuring that questions related to the accuracy or integrity of any part of the work are appropriately investigated and resolved; final approval of the version to be published
IK	Substantial contributions to the conception or design of the work; or the acquisition, analysis, or interpretation of data for the work; drafting the work or revising it critically for important intellectual content; agreement to be accountable for all aspects of the work in ensuring that questions related to the accuracy or integrity of any part of the work are appropriately investigated and resolved; final approval of the version to be published
